# Lithotomy—Bilaterial Operation

**Published:** 1855-03

**Authors:** J. C. Hughes

**Affiliations:** Professor of Surgery


### CASE OF LITHOTOMY—THE BILATERAL OPERATION.

Performed before the Medical Class Nov. 15, 1855,

BY J. C. HUGHES, M. D.,

Professor of Surgery.

Mr. Alexander Shorts, a citizen of Henry county, in this state, had been suffering for more than two years from calculus in the bladder. The history of this case, in a Surgical point of view, presents circumstances of peculiar interest, this being the second operation performed upon him within the last year.

The statement furnished by the patient is as follows: For more than one year previous to the first operation, he had endured much suffering, and during this time had consulted many physicians and submitted to different modes of treatment, without any relief, as well as without obtaining a proper knowledge of his case.

One of his physicians having suggested that there might be stone, and this coinciding with his own views, he was advised to consult Dr. Sanford, of this city, which he did in Nov., 1853. Dr. S. then examined him with a sound for nearly an hour, but was unable to detect stone. The next day he made a second search, but the result was the same. The patient, not satisfied in his own mind, visited Dr. Siveter, of Salem, one of the oldest and most reputable surgeons of our state. Dr. Siveter, after hearing him relate his symptoms, made an examination with the sound, and soon detected stone, upon which he advised an operation. Finding no other means of relief, he determined to have an operation performed, and made his arrangements to visit St. Louis for that purpose. Having reached Keokuk he called upon Dr. Sanford to inform him of his error in diagnosis, when Dr. S. insisted upon his remaining and permitting him to operate. After having examined him again, for the third time, which satisfied him of the presence of stone, the patient consented to remain.

Previous to the operation, the patient stated to Dr. Sanford that he believed there was more than one stone, and gave his reasons, viz: that in his efforts to urinate, the flow of urine would frequently be checked by the presence of a foreign body in the neck of the bladder, and that by the introduction of his finger into the rectum he was enabled to distinctly feel and remove the cause; but no sooner would this be accomplished than another stone of different size would occupy its place. Dr. S. operated upon him, making the lateral incision into the bladder, which is the usual mode. But the operation proved unsuccessful, for having removed but one of the calculi, the symptoms continued of as aggravated a character after the operation as before.

Early in Nov. last, some eight months after the operation alluded to, Mr. Shorts called upon me for my advice in his case. After giving me a full history of the past, which has been briefly narrated, with the symptoms which then presented themselves, I had him taken to the Hospital, where I proceeded to make the necessary examination. In the effort to introduce the sound, I found considerable stricture in the membranous portion of the urethra, as well as a peculiarity with regard to its course. After making an examination by the rectum, I found adhesions existing between that portion of the bowel, and the urethra and neck of the bladder; these adhesions were the result of the former operation, the rectum having unfortunately been cut into. After changing the curve of a small sized sound, I was able to introduce it, and at once discovered stone.

The only chance for relief was from a second operation, which I advised and to which the patient consented. Owing to the adhesions before spoken of, and the dangers which would attend a second operation by the lateral mode, I decided, after consulting with my colleagues, to perform the bilateral operation. By this mode, the safest under the circumstances, I should more certainly avoid any injury to the lower bowel, which would almost inevitably ensue were I to cut in or near the former incision.

Owing to the local as well as general constitutional disturbance produced by the long-continued presence of the calculus, a few days of preparatory treatment were demanded. On the 15th of November he was brought before the medical class for operation.

Having been placed upon the table and properly secured, chloroform was administered, when I proceeded to operate as follows: After the introduction of the staff, which was held by a careful assistant in a perpendicular direction, I commenced by making a semicircular incision with a double edged scalpel from the right side of the median line in the perineum, starting at a point half way between the tuberosity of the ischium and the anus passing half an inch in front of the anus, terminating the incision on the left side of the perineum at a point corresponding with the point at which I started on the right side. Then by several incisions, I divided the superficial fascia, anterior point of the sphincter ani muscle, and cellular tissue, keeping the rectum out of harm's way. The membranous portion of the urethra now being exposed, the groove of the staff was felt for by the nail of the index finger of the left hand, and by this a straight pointed bistoury was inserted into the groove, and made to separate the membranous portion of the urethra for nearly half an inch. Into this opening, I introduced the beak of the double lithotome cache, guided in the same way, keeping the convex surface of the instrument downwards. After pushing it along the groove into the bladder, I withdrew the staff, then turning the lithotome so that its concave surface might present downwards, or towards the anus, I depressed the lever which unsheathed its blades; then by steadily drawing the instrument outwards and downwards, I made a smooth incision through each side of the prostatic portion of urethra and to the depth of some eight lines into each lateral half of the prostate gland.

The instrument now being removed, I was able by the introduction of the left index finger to explore the bladder, and soon found the stone, learned its position and presentation, and by the introduction of the scoop pressing the stone into its hollow, was able to remove it without difficulty. I again introduced the finger and carefully explored the bladder, so that any stone, or fragments might not be overlooked as in the former operation. Satisfied that nothing remained, I proceeded to use the syringe, washing out the viscus thoroughly. A short catheter of large size was then introduced through the wound, which served to conduct the urine, keeping the patient dry, as well as preventing infiltration into the surrounding tissues. In thirty-six hours the catheter was removed, a portion of the urine having already passed through the urethra; on the seventh day the wound was so much closed, that all the urine passed by the natural channel. In ten days the patient was walking about, and on the twelfth day was able to return to his home, a distance of fifty miles.

The stone measured one inch in length, two-thirds of an inch in width, and was two and a quarter inches in circumference. The time occupied in its removal was less than five minutes, and during the operation the patient was entirely free from pain, and enjoying a quiet slumber.

This is the first bilateral operation ever performed in this State, and I confess that the ease and beauty of the operation, and the rapidity of the recovery in this case, have done much towards removing from my mind the prejudices which I had formerly entertained against this mode of operation.

